# First report of eprinomectin-resistant isolates of *Haemonchus contortus* in 5 dairy sheep farms from the *Pyrénées Atlantiques département* in France

**DOI:** 10.1017/S0031182023000069

**Published:** 2023-04

**Authors:** S. Jouffroy, L. Bordes, C. Grisez, J. F. Sutra, T. Cazajous, J. Lafon, N. Dumont, M. Chastel, C. Vial-Novella, D. Achard, H. Karembe, M. Devaux, M. Abbadie, C. Delmas, A. Lespine, P. Jacquiet

**Affiliations:** 1 INTHERES, Université de Toulouse, INRAE, ENVT, 31027 Toulouse Cedex 3, France; 2 IHAP, Université de Toulouse, INRAE, ENVT, 31027 Toulouse Cedex 3, France; 3 CEVA Santé Animale, 33500 Libourne, France; 4 Selarl Vétérinaire du Piémont, 64800 Mirepeix, France; 5 Clinique Vétérinaire du Haut Béarn, 64400 Oloron Ste Marie, France; 6 Vétérinaires Garazi, 64220 St Jean le Vieux, France; 7 Clinique du Saison, 64470 Tardets-Sorholus, France; 8 Centre Départemental Elevage Ovin, 64130 Ordiarp, France

**Keywords:** Dairy sheep, eprinomectin, *Haemonchus contortus*, resistance

## Abstract

Infection of sheep by gastrointestinal nematodes (GIN) in pastoral systems such as those found in the South Western area of France, the *Pyrénées Atlantiques*, is one of the main reasons for economic loss and degradation of their welfare. In the present study, the efficacy of eprinomectin (EPN) was monitored on farms from this area following suspicion of lack of anthelmintic efficacy. Suspicions were raised by veterinarians, based on clinical signs ranging from milk and body condition loss, to anaemia, and mortality. Resistance was evaluated according to the World Association for the Advancement for Veterinary Parasitology (WAAVP) guidelines using fecal egg count reduction tests reinforced by individual analysis of drug concentration in the serum of all treated ewes by high-performance liquid chromatography (HPLC). EPN was administered by subcutaneous (SC) and topical (T) route according to manufacturer's requirements, as well as by the oral route (O) with the topical solution according to off-labelled practices in the field. For the first time in France, the presence of resistant isolates of *Haemonchus contortus* to EPN was observed in 5 dairy sheep farms. The HPLC dosages showed exposure of worms to concentrations compatible with anthelmintic activity for animals treated by the SC and O routes. By contrast, they showed under exposure to the drug of most individuals treated by the T route. EPN is the only null milk withdrawal anthelmintic molecule currently available. The presence of resistant isolates of the pathogenic *H. contortus* to EPN in this important dairy region requires an urgent change in grazing, and sometimes production, systems.

## Graphical abstract

**Figure:**
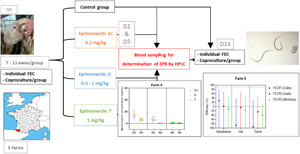

## Introduction

The most south-western *département* of mainland France, the *Pyrénées Atlantiques*, is the country's second largest dairy sheep production area after the Roquefort perimeter. It is where the Protected Designation of Origin (PDO) European labelled cheese Ossau Iraty is made, using milk from 3 local breads: *Manech Tête Rousse*, *Manech Tête Noire* and *Basco Bearnais* sheep. Sheep breeding and grazing for cheese making is a vital part of the local culture and economy, so much so that it has been included in the requirements for the production of PDO Ossau Iraty: ‘ewes should graze for at least 240 days per lactation period’ (INAO, 2015). Locally, the climate is oceanic, with mild temperatures year long and is one of the most humid parts of mainland France, receiving about 1300–1600 mm of rain per year (Meteo France). In this setting, sheep are frequently infected with heavy loads of gastrointestinal nematodes (GIN) when grazing, and farmers have to deal with the challenge of trying to control the parasite load almost all year long. Parasite control has relied on benzimidazoles for several decades, but the use of this family of molecules diminished with the increasing appearance of resistant GIN strains (Geurden *et al*., 2014; Rose Vineer *et al*., 2020) and when the milk withdrawal period changed from zero to at least 4 days in 2014 (Zoetis France, 2009), using these molecules during the lactation period was no longer a financially sound option.

Consequently, the macrocyclic lactone (ML) eprinomectin (EPN) has become the main treatment option during lactation: it has a very low blood to milk partition (Imperiale and Lanusse, 2021), making it the only available molecule in France with a zero milk withdrawal period. First commercialized for cattle as a topical formulation in 1996, and later on as an injectable formulation in 2015 in France, it was not until 2016 and 2020 that EPN was approved for small ruminants, for the topical (HPRA, 2016) and injectable formula, respectively (HPRA, 2020). Before 2016, the topical formulation was administered to dairy sheep and goats off label. Ineffectiveness of the topical formula was rapidly reported in goats on the basis of fecal egg count reduction tests (FECRT) (Murri *et al*., 2014), which prompted veterinarians to use EPN *via* other routes of administration that are known to be associated with higher overall exposure in plasma and tissues and potentially higher efficacy than topical administration (Lespine *et al*., 2012). Lack of efficacy of the topical formula has also been recently described in dairy sheep (Bouy *et al*., 2021; Bordes *et al*., 2022).

Routinely, anthelmintic treatments are administered to the whole lactating flock, usually at a fixed time of the year determined by habit, production stage and/or season of the year. Animals are treated against GIN infections 3–4 times a year on average, using mainly molecules from the ML family (Centre Départemental de l'Elevage Ovin, unpublished data). Together with inaccurate animal weight measurements (underdosing), high frequency of treatment has been proven to be one of the main drivers of anthelmintic resistance (AR) (Wolstenholme *et al*., 2004; Falzon *et al*., 2014; Sangster *et al*., 2018) and loss of efficacy of EPN was expected to happen in the *Pyrénées Atlantiques* sooner or later. From 2018, veterinarians first reported loss of efficacy of avermectins: benzimidazole/ivermectin multi-resistant isolates of *Haemonchus contortus* have been isolated from an ovine meat production farm in the *Hautes Pyrénées* (Cazajous *et al*., 2018) and benzimidazole/EPN multi-resistant isolates of the parasite were identified in a dairy goat herd in the *Pyrénées Atlantiques* (Bordes *et al*., 2020). The implication of this resistance motivated the creation of a 3-year long project, ANTHERIN for ANTHelmintic Resistance in dairy sheep farms: survey and INnovative solutions. The results presented in this study are linked to this project.

Of the 3 main pathogenic species for sheep and goats, *H. contortus* is the most pathogenic and prolific (Arsenopoulos *et al*., 2021). It has probably spread across the globe thanks to commercial activities, and has been able to adapt to different climates (Sallé *et al*., 2019), yet its development remains conditioned by external temperatures and humidity (O'Connor *et al*., 2006; Arsenopoulos *et al*., 2021). *Haemonchus contortus* has also been capable of adapting to anthelmintic treatment, and to this day resistance to all major anthelmintic families have been described (Kotze and Prichard, 2016). Adult worms being blood-sucking parasites, infection of sheep by *H. contortus* causes a range of symptoms depending on host susceptibility and parasite load, from loss of milk production and body condition, to anaemia and death (Arsenopoulos *et al*., 2021).

This study reports for the first time EPN resistance of *H. contortus* in 5 dairy sheep farms in France, investigated between June 2020 and April 2021. In addition to anthelmintic efficacy measured by FECRT, concentrations of EPN were determined in sheep sera 2 and 5 days after treatment, to differentiate cases of loss of efficacy due to drug resistance from those linked to underexposure of GIN to EPN.

## Materials and methods

### Farm selection

Five farms were included in the study based on suspicion of lack of efficacy of EPN in lactating dairy ewes by the veterinary practitioner. These suspicions emerged in February (farm 5), April (farms 1 and 4), June (farm 2) 2020 and April 2021 (farm 3), following oral or injectable EPN treatment. All farmers observed clinical signs compatible with strongylosis that did not improve after EPN treatment in lactating animals. Of these, 3 flocks had symptoms suggestive of haemonchosis (anaemia and on 2 farms mortality), and the remaining 2 flocks showed milk and weight losses. In all cases, the attending veterinarian did a fecal egg count (FEC) about 2 weeks after treatment that revealed the presence of strongyle eggs. Further investigation into the lack of efficacy was conducted to determine whether it was due to underexposure of the strongyles to EPN, or due to the presence of a resistant strain of worms. The 5 investigated farms had an average of 360 lactating ewes [215–500]. Four out of the 5 farms worked with Basco-Bearnaise sheep, and 1 with Manech Tête Rousse (farm 3). All 5 farms sent their lactating ewes in collective middle (1000 m) to high-altitude (⩾2000 m) summer pastures (at least 1 other sheep herd grazing in the same area).

### On-farm protocol

Efficacy of EPN was evaluated using FECRT according to the World Association for the Advancement for Veterinary Parasitology (WAAVP) Guidelines (Coles *et al*., 1992) in lactating dairy ewes. On-farm visits were done rapidly following suspicion, whenever possible. However, for farms 2 and 4, the first Covid-19 lockdown in France caused a 2-month delay between suspicion and visit. On 1 farm (farm 2), the lactating ewes were not available (e.g. they were already grazing in high-altitude summer pastures) and due to the emergency of the situation, FECRT was conducted on ewe lambs. The animals were randomly allocated to 4 groups of 10–11 animals according to their age to compose homogenous groups representative of the herd or the age group. A control group was left untreated, 1 group received injectable EPN (0.2 mg kg^−1^ of LBW, Eprecis^®^injectable, CEVA Santé Animale, Libourne, France; further referred to as the ‘SC group’) and another group received a topical ‘Pour-On’ form of EPN (1 mg kg^−1^ of LBW, Eprinex Multi^®^, Boehringer Ingelheim, Lyon, France; ‘T group’) according to the manufacturer's indication. The last group received EPN orally, using the topical formula (Eprinex Multi^®^, Boehringer Ingelheim; ‘O group’) off label and at the dose of 0.5 mg kg^−1^ of body weight (Badie *et al*., 2015). All animals were treated with a dose rate of 80 kg, which is heavier than the heaviest animal of the group, the 2 encountered breed weights being on average 55–60 kg for female individuals. For the topical treatment, wool was carefully parted so as to apply the solution as well as possible on the skin. For the oral drench, a graduated single-use syringe was used and the absence of regurgitation was verified after treatment. Groups were marked according to their treatment regimen. Feces was collected individually from all animals, samples were identified using the animals' tag 5 digit number and treatment was administered to the pre-defined groups. Fourteen days after treatment, feces was collected individually from all groups. Animals with no fecal samples collected on day 14 after treatment were excluded from the study.

### FECRT

Individual FECs were conducted using the modified McMaster method with a sensitivity of 15 eggs per gram (EpG) (Raynaud *et al*., 1970) within a maximum of 48 h after sampling. Animals for which no strongyle eggs were detected at D0 or animals for which no feces was collected post-treatment were excluded from the study. Once the FEC for the D0 samples were established, the mean FEC was calculated per group, as described by Coles *et al*. (1992). Only groups for which the mean FEC was greater than 300 EPG were included in the study (Cabaret and Berrag, 2004).

Fecal egg count reduction (FECR) and 95% confidence intervals (CIs) were calculated using 3 different formulas, as follows (Coles *et al*., 1992; Dash et al., 1988; McKenna, 2006):
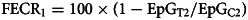

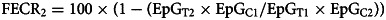

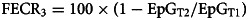
where EpG_T1_ and EpG_T2_ are the arithmetic means of FEC in a treated group at D0 and D14, respectively, and EpG_C1_ and EpG_C2_ are the arithmetic means of FEC in the control group, at D0 and D14, respectively.

CIs for these 3 formulas were calculated according to the methods described by Coles *et al*. (FECR_1_) (Coles *et al*., 1992) and by Lyndal-Murphy *et al.* (FECR_2_ and FECR_3_) (Lyndal-Murphy *et al*., 2014).

The results were interpreted as described in Table 1.

**Table 1.** Interpretation guide for FECRT results (COMBAR, 2021)

Efficacy	Results
Reduced	FECR < 95% *and* lower limit of the 95% CI < 90%
Doubtful	*Either* FECR < 95% *or* lower limit of the 95% CI < 90%
Normal	FECR ⩾ 95% *and* lower limit of the 95% CI ⩾ 90%

### Larvae collection

After FEC, at D0 and D14, stools were combined by group (control, SC, O, T) for fecal culture. Mixing was done so that when the remaining amount after FEC was sufficient, 3–5 g of feces from each animal was combined into the culture. The composite fecal cultures were then incubated for at least 12 days at 24 ± 1°C, and humidified every 2–3 days with tap water. For larvae collection, pots were filled to the brim with tap water and turned up-side down into Petri dishes, which were in turn filled with water. Larvae were collected twice at a 24 h interval in a volume of 40–45 mL and stored vertically at 4°C until DNA extraction (MAFF, 1986).

### Larvae quantification and identification

The supernatant of the tubes stored at 4°C was discarded, and 5 mL of the pellet containing the larvae was kept for further analysis. Furthermore, 500 *μ*L of the pellet was used for the DNA extraction, using the DNeasy PowerSoil kit (QIAGEN, Hilden, Germany). Molecular identification was then performed using a real-time polymerase chain reaction (PCR) according to Milhes *et al*. (2017). Experiments were based on real-time PCR reactions, and standard curves for larval DNA quantitation were established for each PCR run and for 3 species *H. contortus*, *Teladorsagia circumcincta* and *T*richostrongylus *colubriformis.*

### EPN analysis in sheep serum

Blood samples were collected 2 and 5 days post-EPN treatment, in dry tubes from the jugular vein of all treated animals. Blood samples were centrifuged at 3500 rpm for 10 min. Serum was collected and stored at −20°C until further analysis.

After extraction from serum with acetonitrile, EPN concentration was measured using high-performance liquid chromatography with fluorescent detection, as previously described by Sutra *et al*. (1998). The quantification limit of the method was 0.07 ng mL^−1^, and the inter-assay coefficient of variation was lower than 5%.

### Statistical analysis

Graphs were executed using GraphPad Prism version for Windows, GraphPad Software, San Diego, California, USA. Statistical analyses were conducted using R [version 4.1.1 (2021-08-10)] and RStudio version 1.4.1106 (RStudio Team, 2021). Mean EPN concentrations were compared for different treatment regimens within farms using a non-parametric Wilcoxon test.

## Results

### FECRT

Average FEC on D0 in every group and on each farm were above 300 EpG. On farms 2–5, after withdrawing animals not responding to inclusion criteria detailed in ‘FECRT’ section of Materials and methods, 7–11 animals remained per group. At the moment of testing, farm 1 had started the transfer of some animals to summer pastures. The number of animals remaining on the farm was sufficient for 3 groups of 8 lactating ewes (control, treated by subcutaneous and with the topical route) (Table 2).

**Table 2.** Mean FEC results, with minimal and maximal individual value and final number of animals included, per group and per farm

Farm		Control		SC		O		T	
		D0	D14	D0	D14	D0	D14	D0	D14
1	Mean	**850**	1956	**2019**	2689			**1408**	919
	Min–Max	**100–2250**	0–7100	**100–10 000**	0–11 750			**150–5300**	50–4800
	N	8		8				8	
2^1^	Mean	**3028**	3756	**4036**	486	**1445**	400	**5072**	394
	Min–Max	**75–6550**	300–7550	**800–17 200**	100–2000	450–2750	0–1100	**850–12 700**	50–950
	N	8		11		10		9	
3	Mean	**1428**	800	**662**	650	**1089**	1039	**1205**	885
	Min–Max	**100–6550**	50–2250	**15–2050**	0–1950	50–2550	0–2550	**50–2350**	0–2200
	N	9		10		9		10	
4	Mean	**1693**	194	**1813**	302	**1986**	50	**1338**	581
	Min–Max	**100–8500**	45–500	**150–5300**	0–2000	150–8200	0–200	**50–3250**	50–1300
	N	7		8		7		8	
5	Mean	**469**	525	**376**	413	**838**	980	**735**	1020
	Min–Max	**50–1550**	0–1800	**60–1700**	0–1600	50–2650	100–2700	**350–1200**	300–1900
	N	10		8		10		10	

In bold, values of mean and minimum–maximum FEC on day 0, for an easier quick reading.

1 On farm 2, FECRT were conducted on ewe lambs.

**Fig. 1.**
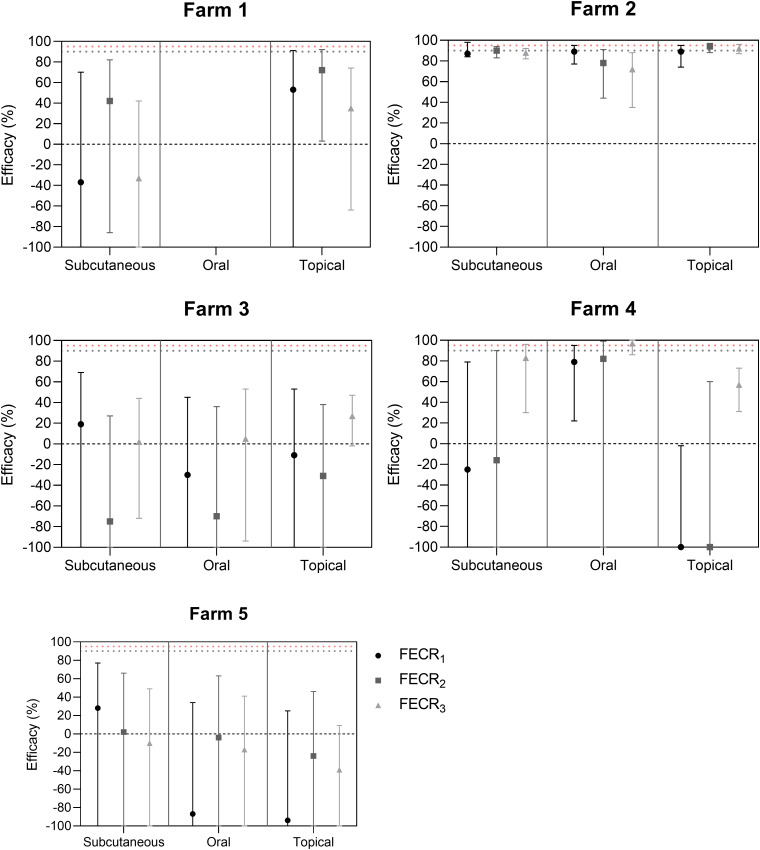
FECR results and confidence intervals for the 5 farms, calculated according to 3 different formulas and for all treatment types.

Calculated FECR are presented in Fig. 1. Depending on the farm, the group and the formula used, FECR results varied widely. All values of FECR were lower than 95%, except for FECR_3_ for group O of farm 4 and all lower level CIs were inferior to 90%. With the exception of FECR_3_ of group O for farm 4 (97%), these criteria indicate reduced efficacy for all 5 farms. Regarding group O of farm 4, interpretation would have been that efficacy of EPN was doubtful. However, given results obtained with 2 other formulas, including FECR_1_ recommended by the WAAVP guidelines, efficacy for group O of farm 4 is clearly reduced.

Different FECR formulas yield different results, depending mainly on the value of the mean FEC of different groups. FECR_1_ and FECR_3_ yield similar results when the mean FEC of the control group on D14 is close to the mean FEC of treated groups on D0, as is the case on farm 2. On this farm, the FEC on D14 for the control group (3756 EpG) is not significantly different from FECs for the SC, O and T groups (respectively 4036, 1445 and 5072 EpG) on D0 (*P* value < 0.05). FECR_1_ formula that compares the FEC of treated and control groups on D14 yields similar percentages to FECR_3_, which compares FEC of treated group on D14 and D0, in this case (e.g. 87 and 88% reduction for the SC group with FECR_1_ and FECR_3_, respectively). On farm 4, percentages of fecal egg reduction differ between FECR_1_ and FECR_3_. There is a significant difference between the FEC of the control group on D0 (194 EpG) and of the treated groups on D14 (1813, 1986 and 1338 EpG for SC, O and T groups, respectively, *P* < 0.05). For example, considering the SC group on this farm, post-treatment fecal egg reduction is −25% using FECR_1_, yet it is 83% using FECR_3_.

**Fig. 2.**
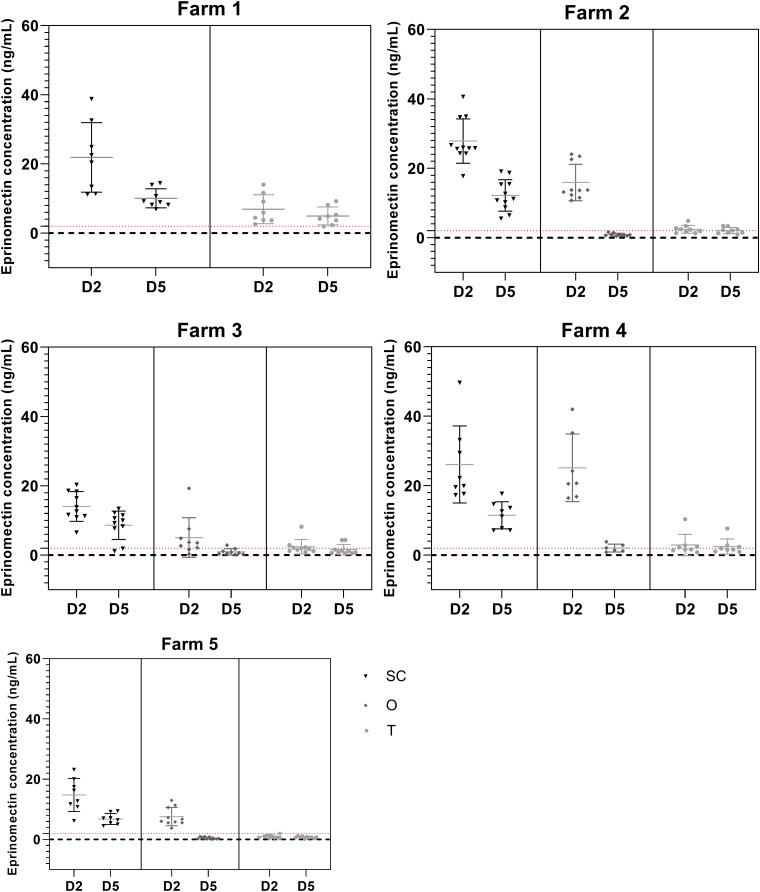
Serological concentrations of EPN by farm, and by administration route. Horizontal lines indicate mean concentrations per treatment type and per day post-treatment. Black triangle: SC group; medium grey diamond: O group; light grey dot: T group. Red dotted line is at 2 ng mL^−1^. D2: 2 days after treatment; D5: 5 days after treatment. Individual concentrations and mean concentrations per group ± s.d. (7–11 animals per group).

Mean EPN concentrations in serum 2 days after administration were significantly higher after subcutaneous injection than after oral administration in 3 out of the 4 farms where both these routes were tested (Fig. 2; farms 2, 3 and 5; *P* value < 0.01). On farm 4, there was no significant difference between both routes. Topical administration of EPN resulted in dramatically low drug concentrations in the serum of ewes in all 5 farms (Fig. 2; *P* value < 0.01). Mean values were between 14.35 [s.d.: 4.43] and 27.84 ng mL^−1^ [s.d.: 6.37] for the SC group; 5.06 [s.d.: 5.71] and 25.14 ng mL^−1^ [s.d.: 9.74] for the O group and between 0.97 [s.d.: 0.54] and 6.92 ng mL^−1^ [s.d.: 4.2] for the T group, 2 days after treatment (Fig. 2). Five days after treatment, EPN serological concentrations were significantly higher after subcutaneous injection than after either of the other routes.

For the T groups, the individual concentrations of EPN varied from being all below 2 ng mL^−1^ (farm 5) to all above (farm 1). On farms 2, 3 and 4, respectively, 44, 60 and 50% of individual EPN concentration values in the T group were below this concentration threshold (Fig. 2 and Supplementary data).

### Strongyle species present pre- and post treatment

Before treatment, at D0, the strongyle species most present in the cultured feces of every farm was *H. contortus*. On farm 1, it was the only species of the 3 the qPCR could identify. On the 4 other farms, *T. colubriformis* and *T. circumcincta* were present in small proportions: *T. colubriformis* larvae composed at most 16.4% (on farm 3) of the larval culture yield (Table 3).

**Table 3.** Proportions (%) of the 3 main pathogenic species in bulk fecal cultures before (D0) and after (D14) for every group of the 5 farms

Farm	Control						SC						O						T					
	D0			D14			D0			D14			D0			D14			D0			D14		
	*Hc*	*Te*	*Tr*	*Hc*	*Te*	*Tr*	*Hc*	*Te*	*Tr*	*Hc*	*Te*	*Tr*	*Hc*	*Te*	*Tr*	*Hc*	*Te*	*Tr*	*Hc*	*Te*	*Tr*	*Hc*	*Te*	*Tr*
1	100	0	0	100	0	0	100	0	0	100	0	0							100	0	0	100	0	0
2^a^	97.4	2.2	0.4	99.2	0	0.8	99.3	0.6	0.2	100	0	0	97.8	0.4	1.8	0	0	100	98.6	0.7	0.7	92.1	0	7.9
3	94.5	1.2	4.3	88.8	4.6	6.6	99.4	0.6	0	65.8	0	34.2	78.2	15.2	6.7	100	0	0	78.7	4.8	16.4	85.7	4.1	10.2
4	75.5	12.2	12.4	100	0	0	88.8	0.3	10.9	100	0	0	78.8	8.9	12.3	100	0	0	56.1	0.1	43.8	100	0	0
5	99.9	0.1	0	100	0	0	99.8	0	0	100	0	0	100	0	0	100	0	0	99.4	0	0.6	90.6	1	8.4

*Hc*, *Haemonchus contortus*; *Te*, *Teladorsagia circumcincta*; *Tr*, *Trichostrongylus colubriformis*; SC, subcutaneous EPN; O, oral drench EPN; T, topical EPN

^a^On farm 2, FECRT were conducted on ewe lambs.

After treatment, *H. contortus* was the main species identified on the 5 farms (Table 3). For 4 farms out of 5, *H. contortus* was the only species remaining for the groups treated by sub-cutaneous injection. On farm 3, *T. colubriformis* larvae were also present in the cultures post injectable EPN treatment (Table 3). On farm 2, *T. colubriformis* larvae were the only species present post oral treatment, however in small quantities (Supplementary data, Table S8). *Haemonchus contortus* and *T. colubriformis* larvae were present in the post-treatment fecal cultures of the ewes treated with a topical solution, except in farm 1 where the collected larvae were 100% *H. contortus*.

## Discussion

In this field study, EPN was found to have a reduced efficacy whatever the formula tested (SC, O, T) in 5 commercial dairy sheep farms where veterinarians and farmers suspected a lack of efficacy based on the persistence of clinical signs following oral or injectable treatment. Low drug efficacy was observed even in the SC administration group, which showed the highest drug levels in the hosts' serum.

We confirmed that EPN serum levels are highly dependent upon the route of administration. EPN concentration measured in the serum of treated lactating and pre-lactating ewes 2 days after treatment was well above the 2 ng mL^−1^ minimal efficacy concentration for all the animals treated with a subcutaneous or oral formula, which supports the idea that these ewes received a dose of the molecule that can be considered sufficient to kill strongyle adults. The poor FECRT performance obtained in these animals strongly supports the presence of strongyles resistant to EPN. Although no pharmacokinetic and pharmodynamic (PK/PD) study has been conducted to specifically identify the minimal EPN therapeutic dose, the minimal active dose for this drug family (e.g. ivermectin) has been shown to be above 2 ng mL^−1^ (Bousquet-Mélou *et al*., 2011). This concentration is therefore considered a threshold that guaranties efficacy of EPN in small ruminants (Hoste *et al*., 2004; Rostang *et al*., 2020). The time at which EPN concentration reaches its highest averages between 1.2(±0.4) and 3.13(±2.99) days depending on dosage rate and physiology of the animals was considered (Imperiale *et al*., 2006; Hodošček *et al*., 2008; Hamel *et al*., 2017). Average serum EPN concentrations in the groups treated with a topical solution of EPN were low in most of the farms on D2. Differences in the breed and physiology of the animals could explain values below those found by Hamel *et al*. (2017) in dry merino crossed sheep. In the present study, on all farms except farm 2, FECR tests were conducted on lactating ewes and in all farms animals bore a substantial worm burden, and both lactation and body condition have been shown to influence ML pharmacokinetic parameters (Lespine *et al*., 2004, 2012; Rostang *et al*., 2020).

The purpose of this study was to provide reliable information about AR status in farms. We set up a feasible protocol to monitor drug efficacy through combining FECR and drug concentration monitoring in treated animals. Measuring concentrations at 2 critical times 2 days (close to maximal concentration) and 5 days (elimination phase), these data points allow simulation of the complete drugs' pharmacokinetics.

FECR values indicate a reduced efficacy with all 3 formulas and the low FECR after a topical treatment is due to the presence of resistant strongyle. However, cases of underexposure of GIN to EPN when using a topical formula have previously been reported by veterinary practitioners and confirmed by 2 recent studies in France. Bouy *et al*. and Bordes *et al*. described cases where FECR after EPN treatment were below 95% when using the topical solution and above 95% when animals of the same flock were treated with a subcutaneous solution (Bouy *et al*., 2021; Bordes *et al*., 2022). In the study by Bordes *et al*. (2022) serum concentrations of EPN were below 2 ng mL^−1^ for all animals treated with a topical formula. These findings are in line with others (Hoste *et al*., 2004; Hodošček *et al*., 2008) that underline the highly variable bioavailability of the topical formula of EPN. The use of topical route for EPN is therefore not recommended.

The significant difference between mean concentrations of EPN in SC and T groups is observed although the dose rate for the topical solution is 5 times higher than for the injection solution (1 mg kg^−1^ of LBW for the topical solution and 0.2 mg kg^−1^ LBW for the injection solution). Given the impact of ML on non-target species such as dung beetles, elimination of the molecule, through direct contact or by the fecal matters, could have an impact on pasture quality (Sands and Wall, 2018; Verdú *et al*., 2018; Weaving *et al*., 2020).

Upon communication of these results back to farmers and veterinarians, one of the challenges was explaining the discrepancy between the reduction calculated for the SC group and the one calculated for the oral drench group on the same farm at the same date, therefore with the same control group. Questions arose for these 2 treatment regimens although not for the topical formula group for the reasons explained above. Much has already been said about FECRT and their limits, and the debate remains open as summarized very recently by Morgan *et al*. (2022). However, our field experience teaches us that such differences need explanations, in order to be accepted by farmers and veterinarians. In our study, FECRT variations could hardly be explained by pharmacological factors, as EPN concentrations were well above 2 ng mL^−1^, a concentration at which the molecule is considered to be efficient (Guillot *et al*., 1986; Guyonnet *et al*., 2017). Although ewes of similar ages were evenly distributed between the SC and O group, differences in mean EPG partly explain the differences observed between the FECR of these 2 treatment formulas. Host factors, such as consistency of feces could contribute to variations in egg outputs and contribute to variations from one group to another, and host immunity could also be of importance for the inter-individual variations. *Haemonchus contortus* being the main strongyle species before and after treatment in all 5 farms, variations in FECR due to initial diversity of species is limited. Parasite fitness, including its fecundity, could add to the observed variations, although this should be limited by the fact that it is highly probable all sheep of the same flock harbour the same resistant isolate.

Identification of the 3 main pathogenic strongyle species for small ruminants was done using fecal cultures and qPCR. In all 5 farms, *H. contortus* was the main species present after treatment. Furthermore, resistance to EPR of isolates from farms 1 and 4 was confirmed by infestation and EPN challenge in experimental sheep (G. Salle, unpublished data). Interestingly, before treatment, *H. contortus* was predominant in the fecal culture yields of all 5 farms, and on farm 1 it was the only species present. These farms were investigated because EPN treatments were not resolving the observed symptoms, although the farmers had sometimes drenched the animals with EPN several times before calling their veterinarians to alert them to the problem. Our hypothesis is that these repeated treatments have exerted an important selective pressure upon the present worms, and cleared all susceptible populations. Farms 1, 4 and 5 were included in this study because during the spring of 2020 they were facing dire situations. On farms 2 and 3, health issues were sub-acute. Veterinarians knew the history of these 5 farms and that they are prone to facing GIN issues. This first report of EPN resistance in dairy sheep in this dairy region is a description of how resistance can manifest itself and how it has been investigated. This study was conducted to investigate issues raised by some farmers and veterinarians, and the aim was not to determine the prevalence of EPN resistance. Hence, to this date, it is not known if these cases are the tip of the iceberg regarding EPN resistance in the *Pyrénées Atlantiques*, and if they are, how big the iceberg is.

In post topical treatment coprocultures from farms 2 to 5, *T. colubriformis* and *T. circumcincta* (on farm 3) were also identified. Considering the EPR serum concentration for this treatment formula, we hypothesize these worms are still present because they have been under-exposed to the molecule. Furthermore, the intestinal species *T. colubriformis* has already been described as dose-limiting for EPN, its presence after topical treatment is not surprising (Chartier *et al*., 1999; Hoste *et al*., 2004).

In this southwestern area of France, resistance of *H. contortus* to EPN had previously been described in lactating goats (Bordes *et al*., 2020) and in sheep raised for meat (Cazajous *et al*., 2018), but this is the first report of the presence of resistant strongyle strains in dairy sheep in the *Pyrénées Atlantiques*. Special attention has been brought to EPN resistance in this area, as well as in the Roquefort area, being the only molecule with a zero milk withdrawal period. Hence, EPN resistance means treating ewes against GIN during their lactation will inevitably come at the cost of, at least, throwing away milk during the withdrawal period. Farmers have to find other ways of controlling parasitism than solely relying on drugs, in order to keep their grazing flocks healthy while maintaining a decent level of production. The presence of resistant strains is also of concern here because of the use of collective pastures that could allow for the dissemination of resistant strains.

Resistance of GIN to ML molecules in France appeared relatively late, compared to other countries: Geurden *et al*. (2014) and Paraud *et al*. (2010) reported no resistance to ML in the 2 main dairy sheep regions and in dairy goats, respectively, and the first case of ivermectin resistance was described for *T. circumcincta* in meat sheep production in central France in 2014 (Paraud *et al*., 2016). These observations are in line with the general trend in Europe of increasing AR throughout anthelmintic families and GIN species (Rose Vineer *et al*., 2020). Of the 3 main pathogenic GIN species for sheep, *H. contortus*, originally a parasite of warm and humid climates (Sallé *et al*., 2019; Arsenopoulos *et al*., 2021), profits from global warming and is reaching farther north in Europe and infection pressure expands during the year (Rose *et al*., 2016). Although located in southern France, the area of interest for the present study harbours an oceanic climate with normally mild temperatures. In the last few years, it seems to be hosting more frequent cases of severe haemonchosis (P. Jacquiet, T. Cazajous, personal communication), in line with the rise of average temperatures. Four of the 5 cases of haemonchosis presented here happened after a particularly mild winter (MeteoFrance, 2020), which could have allowed an increased survival of *H. contortus* larvae on pastures. As well as adapting to climate, *H. contortus* has also been adapting to ML treatments, and in addition to the present study, resistance of the Barber Pole Worm has also been recently described in sheep in southwest England (Bull *et al*., 2022) and in goats in Austria (Hinney *et al*., 2022).

## Conclusion

The present study reports for the first time in France 5 cases of EPN-resistant *H. contortus* in dairy ewes in the south western *département* of the *Pyrénées Atlantiques*, the country's second largest sheep milk and cheese production area. These isolated cases are particularly worrying for the dairy sheep production in this area, where an important percentage of farms rely on summer pastures, usually shared, as part of their forage resource. However, they reflect what is happening elsewhere in Europe, in meat but also dairy sheep production. For milk-producing farms, EPN resistance in GIN does not come as a complete surprise, as only a very small pool of molecules is used for treatment. This study further confirms the variability of EPN serum concentration depending on administration route, with the injectable formula yielding the highest concentration post-treatment. The topical solution yields highly variable and sub-therapeutic EPN concentrations. Therefore, the use of topical EPN should be discouraged so as not to exacerbate the problem of resistance to ML and to better maintain good animal health. Dairy production adds an extra challenge to field management, yet some farmers have already found some encouraging, and hopefully durable, improvements to their system. For the farms not yet facing resistance of GIN to EPN, a simple and robust protocol to target and selectively treat lactating ewes to maintain a refuge population is in trial.

## Data Availability

Data supporting results are provided within the article and in the supplementary materials.

## Supplementary material

For supplementary material accompanying this paper visit https://doi.org/10.1017/S0031182023000069. click here to view supplementary material
