# Supplementary material for: First report of eprinomectin-resistant isolates of Haemonchus contortus in 5 dairy sheep farms from the Pyrénées Atlantiques département in France
Source: Parasitology. 2023 Jan 20;150(4):365–73. doi: 10.1017/S0031182023000069 (PMC10090475; doi:10.1017/S0031182023000069)
Supplement: Supplementary file 1 [file S0031182023000069sup001.docx]

**Supplementary data 1** : individual values of pre (D0) and post (D14) treatment and eprinomectin concentration (ng/mL), sorted by group.

| Farm | Formula | D0 FEC | D14 FEC | EPN D2 | EPN D5 |
| --- | --- | --- | --- | --- | --- |
| 1 | Control | 2250 | 7100 |  |  |
|  | Control | 1250 | 5600 |  |  |
|  | Control | 1150 | 2300 |  |  |
|  | Control | 950 | 0 |  |  |
|  | Control | 600 | 50 |  |  |
|  | Control | 350 | 0 |  |  |
|  | Control | 150 | 0 |  |  |
|  | Control | 100 | 600 |  |  |
|  | SC | 10000 | 11750 | 11,22 | 6,93 |
|  | SC | 3100 | 1800 | 11,42 | 9,23 |
|  | SC | 1650 | 5050 | 13,41 | 8,95 |
|  | SC | 600 | 2700 | 32,59 | 8,20 |
|  | SC | 400 | 100 | 20,41 | 14,46 |
|  | SC | 150 | 15 | 38,79 | 13,96 |
|  | SC | 150 | 0 | 24,92 | 10,69 |
|  | SC | 100 | 100 | 22,52 | 8,18 |
|  | Topical | 5300 | 4800 | 3,70 | 2,35 |
|  | Topical | 1850 | 300 | 11,64 | 8,14 |
|  | Topical | 1600 | 350 | 14,03 | 4,92 |
|  | Topical | 1400 | 50 | 5,93 | 5,28 |
|  | Topical | 500 | 750 | 4,47 | 4,16 |
|  | Topical | 300 | 150 | 2,58 | 1,97 |
|  | Topical | 165 | 700 | 3,82 | 3,73 |
|  | Topical | 150 | 250 | 9,15 | 9,22 |

**Supplementary data 2** : individual values of pre (D0) and post (D14) treatment and eprinomectin concentration (ng/mL), sorted by group.

| Farm | Formula | D0 FEC | D14 FEC | EPN D2 | EPN D5 |
| --- | --- | --- | --- | --- | --- |
| 2 | Control | 6550 | 7000 |  |  |
|  | Control | 6000 | 7200 |  |  |
|  | Control | 1050 | 1450 |  |  |
|  | Control | 2850 | 3150 |  |  |
|  | Control | 1400 | 1400 |  |  |
|  | Control | 75 | 300 |  |  |
|  | Control | 4950 | 7550 |  |  |
|  | Control | 1350 | 2000 |  |  |
|  | SC | 4000 | 100 | 34,71 | 15,31 |
|  | SC | 800 | 100 | 25,81 | 11,19 |
|  | SC | 1450 | 200 | 24,28 | 19,07 |
|  | SC | 1500 | 250 | 26,66 | 10,72 |
|  | SC | 4650 | 100 | 24,32 | 18,70 |
|  | SC | 1200 | 200 | 25,60 | 10,22 |
|  | SC | 1250 | 100 | 40,61 | 6,43 |
|  | SC | 2100 | 800 | 34,90 | 15,41 |
|  | SC | 4900 | 850 | 17,73 | 8,74 |
|  | SC | 17200 | 650 | 25,92 | 12,66 |
|  | SC | 5350 | 2000 | 25,72 | 5,50 |
|  | Oral | 550 | 1100 | 12,34 | 0,93 |
|  | Oral | 1600 | 300 | 13,18 | 0,90 |
|  | Oral | 1850 | 350 | 13,73 | 0,71 |
|  | Oral | 800 | 750 | 22,58 | 0,84 |
|  | Oral | 450 | 0 | 13,65 | 0,45 |
|  | Oral | 1200 | 50 | 11,53 | 0,52 |
|  | Oral | 1800 | 50 | 10,75 | 0,66 |
|  | Oral | 2150 | 550 | 13,77 | 0,56 |
|  | Oral | 1300 | 400 | 24,07 | 1,62 |
|  | Topical | 850 | 50 | 1,33 | 1,42 |
|  | Topical | 3250 | 550 | 1,65 | 1,00 |
|  | Topical | 1800 | 250 | 2,43 | 1,25 |
|  | Topical | 5100 | 950 | 1,84 | 2,05 |
|  | Topical | 1100 | 400 | 4,83 | 3,41 |
|  | Topical | 11950 | 800 | 3,38 | 3,32 |
|  | Topical | 6400 | 350 | 2,29 | 2,23 |
|  | Topical | 2500 | 100 | 2,71 | 1,48 |
|  | Topical | 12700 | 100 | 1,32 | 2,35 |

**Supplementary data 3**: individual values of pre (D0) and post (D14) treatment and eprinomectin concentration (ng/mL), sorted by group.

| Farm | Formula | D0 FEC | D14 FEC | EPN D2 | EPN D5 |
| --- | --- | --- | --- | --- | --- |
| 3 | Control | 6550 | 2250 |  |  |
| 3 | Control | 1200 | 800 |  |  |
|  | Control | 1200 | 550 |  |  |
|  | Control | 100 | 50 |  |  |
|  | Control | 150 | 350 |  |  |
|  | Control | 1050 | 600 |  |  |
|  | Control | 400 | 550 |  |  |
|  | Control | 300 | 150 |  |  |
|  | Control | 1900 | 1900 |  |  |
|  | SC | 400 | 200 | 20,32 | 11,45 |
|  | SC | 2050 | 1750 | 18,34 | 13,41 |
|  | SC | 150 | 1150 | 11,25* | 9,96 |
|  | SC | 750 | 400 | 12,6 | 12,22 |
|  | SC | 50 | 0 | 16,42 | 10,61 |
|  | SC | 15 | 0 | 10,92 | 1,89 |
|  | SC | 50 | 50 | 18,56 | 7,73 |
|  | SC | 700 | 650 | 6,54 | 1,13 |
|  | SC | 1700 | 1950 | 11,61 | 8,28 |
|  | SC | 750 | 350 | 13,86 | 9,22 |
|  | Oral | 2550 | 2550 | 0,29 | 1,83 |
|  | Oral | 1900 | 0 | 19,26 | 2,85 |
|  | Oral | 350 | 1750 | 7,57 | 0,83 |
|  | Oral | 750 | 750 | 3,49 | 0,48 |
|  | Oral | 1900 | 1300 | 1,59 | 0,17 |
|  | Oral | 50 | 0 | 4,84 | 1,08 |
|  | Oral | 450 | 900 | 2,15 | 0,35 |
|  | Oral | 1650 | 1650 | 2,66 | 0,64 |
|  | Oral | 200 | 450 | 3,73 | 0,24 |
|  | Topical | 2150 | 1500 | 2,23 | 4,31 |
|  | Topical | 1500 | 1000 | 8,19 | 0,83 |
|  | Topical | 50 | 150 | 1,97 | 1,21 |
|  | Topical | 750 | 750 | 2,15 | 1,22 |
|  | Topical | 1050 | 850 | 2,87 | 1,56 |
|  | Topical | 50 | 0 | 1,73 | 4,37 |
|  | Topical | 1750 | 450 | 1,12 | 0,56 |
|  | Topical | 150 | 100 | 0,96 | 0,54 |
|  | Topical | 2350 | 1850 | 1,21 | 0,58 |
|  | Topical | 2250 | 2200 | 1,52 | 0,78 |

**Supplementary data 4**: individual values of pre (D0) and post (D14) treatment and eprinomectin concentration (ng/mL), sorted by group.

| Farm | Formula | D0 FEC | D14 FEC | EPN D2 | EPN D5 |
| --- | --- | --- | --- | --- | --- |
| 4 | Control | 100 | 350 |  |  |
| 4 | Control | 8500 | 100 |  |  |
|  | Control | 400 | 450 |  |  |
|  | Control | 300 | 500 |  |  |
|  | Control | 150 | 50 |  |  |
|  | Control | 550 | 200 |  |  |
|  | Control | 1850 | 45 |  |  |
|  | SC | 1850 | 150 | 33,17 | 7,18 |
|  | SC | 1200 | 50 | 49,66 | 14,67 |
|  | SC | 1450 | 50 | 29,40 | 11,12 |
|  | SC | 5300 | 2000 | 19,58 | 17,67 |
|  | SC | 3500 | 100 | 17,28 | 7,77 |
|  | SC | 500 | 50 | 19,91 | 13,57 |
|  | SC | 550 | 15 | 22,20 | 12,62 |
|  | SC | 150 | 0 | 17,64 | 7,11 |
|  | Oral | 3050 | 0 | 20,71 | 0,83 |
|  | Oral | 200 | 50 | 35,17 | 1,16 |
|  | Oral | 8200 | 100 | 20,58 | 2,09 |
|  | Oral | 150 | 0 | 24,22 | 1,41 |
|  | Oral | 1250 | 200 | 16,48 | 3,86 |
|  | Oral | 800 | 0 | 16,84 |  |
|  | Oral | 250 | 0 | 41,99 | 2,86 |
|  | Topical | 950 | 650 | 2,44 | 1,60 |
|  | Topical | 2700 | 1250 | 2,24 | 2,40 |
|  | Topical | 3250 | 1300 | 0,78 | 0,96 |
|  | Topical | 50 | 50 | 1,56 | 1,86 |
|  | Topical | 1150 | 700 | 1,63 | 1,55 |
|  | Topical | 1550 | 200 | 10,33 | 7,64 |
|  | Topical | 750 | 300 | 2,83 | 2,83 |
|  | Topical | 300 | 200 | 1,36 | 1,02 |

**Supplementary data 5**: individual values of pre (D0) and post (D14) treatment and eprinomectin concentration (ng/mL), sorted by group.

| Farm  5 | Formula | D0 FEC | D14 FEC | EPN D2 | EPN D5 |
| --- | --- | --- | --- | --- | --- |
|  | Control | 900 | 200 |  |  |
|  | Control | 650 | 900 |  |  |
|  | Control | 100 | 0 |  |  |
|  | Control | 50 | 400 |  |  |
|  | Control | 250 | 700 |  |  |
|  | Control | 200 | 100 |  |  |
|  | Control | 1550 | 1800 |  |  |
|  | Control | 50 | 100 |  |  |
|  | SC | 200 | 100 | 17,43 | 5,02 |
|  | SC | 1700 | 1600 | 23,18 | 9,43 |
|  | SC | 150 | 200 | 12,73 | 5,43 |
|  | SC | 450 | 300 | 10,83 | 7,01 |
|  | SC | 250 | 100 | 6,16 | 6,42 |
|  | SC | 100 | 600 | 16,23 | 9,19 |
|  | SC | 100 | 0 | 11,74 | 7,13 |
|  | SC | 60 | 400 | 20,02 | 4,48 |
|  | Oral | 2350 | 2700 | 5,54 | 0,69 |
|  | Oral | 75 | 200 | 6,01 | 0,31 |
|  | Oral | 50 | 100 | 10,60 | 0,93 |
|  | Oral | 550 | 800 | 7,27 | 0,25 |
|  | Oral | 1200 | 200 | 6,76 | 0,20 |
|  | Oral | 2650 | 1000 | 5,84 | 0,17 |
|  | Oral | 500 | 700 | 13,01 | 0,82 |
|  | Oral | 250 | 300 | 11,36 | 1,00 |
|  | Oral | 300 | 1200 | 5,55 | 0,19 |
|  | Oral | 450 | 2600 | 3,75 | 0,15 |
|  | Topical | 600 | 500 | 1,08 | 0,73 |
|  | Topical | 700 | 1900 | 1,98 | 1,20 |
|  | Topical | 1200 | 1900 | 0,67 | 0,89 |
|  | Topical | 750 | 400 | 0,35 | 0,29 |
|  | Topical | 450 | 300 | 0,31 | 0,16 |
|  | Topical | 800 | 1900 | 0,74 | 0,75 |
|  | Topical | 350 | 500 | 1,47 | 1,15 |
|  | Topical | 650 | 600 | 0,55 | 0,72 |
|  | Topical | 1100 | 1700 | 1,36 | 0,65 |
|  | Topical | 750 | 500 | 1,20 | 0,60 |

Supplementary data 6: FECR results and Confidence Intervals (CI) for the 5 farms, calculated according to 3 different formulas and for all treatment type. SC: subcutaneous EPN; O : oral drench EPN and T : topical EPN.

|  | **FECR_1_**  (Coles *et al.*, 1992) | | | **FECR_2_**  (Dash *et al.*, 1988) | | | **FECR_3_**  (McKenna, 2006) | | |
| --- | --- | --- | --- | --- | --- | --- | --- | --- | --- |
| Farm number | **SC** | **O** | **T** | **SC** | **O** | **T** | **SC** | **O** | **T** |
|  | [CI] | [CI] | [CI] | [CI] | [CI] | [CI] | [CI] | [CI] | [CI] |
| 1 | **-37** |  | **53** | **42** |  | **72** | **-33** |  | **35** |
|  | [-530 ; 70] |  | [-141 ; 91] | [-86 ; 82] |  | [3 ; 92] | [-205 ; 42] |  | [-64 ; 74] |
| 2 | **87** | **89** | **89** | **90** | **78** | **94** | **88** | **72** | **92** |
|  | [84 ; 98] | [77 ; 95] | [74 ; 95] | [83 ; 94] | [44 ; 91] | [88 ; 97] | [82 ; 92] | [35 ; 88] | [87 ; 96] |
| 3 | **19** | **-30** | **-11** | **-75** | **-70** | **-31** | **2** | **5** | **27** |
|  | [-115 ; 69] | [-207 ; 45] | [-161 ; 53] | [-321 ; 27 ] | [-356 ; 36] | [-177 ; 38] | [-72 ; 44] | [-94 ; 53] | [-2 ; 47] |
| 4 | **-25** | **79** | **-140** | **-16** | **82** | **-204** | **83** | **97** | **57** |
|  | [-624 ; 79] | [22 ; 95] | [-466 ; -2] | [-1239 ; 90] | [-153 ; 99] | [-2219 ; 60] | [30 ; 96] | [86 ; 100] | [31 ; 73] |
| 5 | **28** | **-87** | **-94** | **2** | **-4** | **-24** | **-10** | **-17** | **-39** |
|  | [-169 ; 77] | [-431 ; 34] | [-403 ; 25] | [-184 ; 66] | [-193 ; 63] | [-183 ; 46] | [-136 ; 49] | [-132 ; 41] | [-112 ; 9] |

Supplementary data 7 : Larvae count from larval cultures before treatment on the 5 farms. N : larval ; percentages give the relative proportions of the 3 species per group.

| Farm | Formula | *Haemonchus contortus* | | *Teladorsagia circumcincta* | | *Trichostrongylus colubriformis* | |
| --- | --- | --- | --- | --- | --- | --- | --- |
|  |  | N | % | N | % | N | % |
| **1** | Control | 1853 | 100 | 0 | 0 | 0 | 0 |
|  | *SC* | *1023* | *100* | *0* | *0* | *0* | *0* |
|  |  |  |  |  |  |  |  |
|  | **T** | **368** | **100** | **0** | **0** | **0** | **0** |
| **2** | Control | 2685 | 97,4 | 62 | 2,2 | 11 | 0,4 |
|  | *SC* | *6241* | *99,3* | *37* | *0,6* | *10* | *0,2* |
|  | O | 1725 | 97,8 | 7 | 0,4 | 32 | 1,8 |
|  | **T** | **4100** | **98,6** | **30** | **0,7** | **28** | **0,7** |
| **3** | Control | 480 | 94,5 | 6 | 1,2 | 22 | 4,3 |
|  | *SC* | *888* | *99,4* | *5* | *0,6* | *0* | *0* |
|  | O | 4383 | 78,2 | 852 | 15,2 | 373 | 6,7 |
|  | **T** | **13617** | **78,7** | **836** | **4,8** | **2841** | **16,4** |
| **4** | Control | 23704 | 75,5 | 3830 | 12,2 | 3882 | 12,4 |
|  | *SC* | *5891* | *88,8* | *20* | *0,3* | *724* | *10,9* |
|  | O | 632 | 78,8 | 71 | 8,9 | 99 | 12,3 |
|  | **T** | **12017** | **56,1** | **28** | **0,1** | **9381** | **43,8** |
| **5** | Control | 2689 | 99,9 | 2 | 0,1 | 0 | 0 |
|  | *SC* | *627* | *99,8* | *1* | *0,2* | *0* | *0* |
|  | O | 1179 | 100 | 0 | 0 | 0 | 0 |
|  | **T** | **7098** | **99,4** | **0** | **0** | **45** | **0,6** |

Supplementary data 8: Larvae count from larval cultures after treatment on the 5 farms. N : larval ; percentages give the relative proportions of the 3 species per group.

| **Farm** | Formula | *Haemonchus contortus* | | *Teladorsagia circumcincta* | | *Trichostrongylus colubriformis* | |
| --- | --- | --- | --- | --- | --- | --- | --- |
|  |  | N | % | N | % | N | % |
| **1** | Control | 2410 | 100 | 0 | 0 | 0 | 0 |
|  | *SC* | *11915* | *100* | *0* | *0* | *0* | *0* |
|  |  |  |  |  |  |  |  |
|  | **T** | **728** | **100** | **0** | **0** | **0** | **0** |
| **2** | Control | 14363 | 99,2 | 1 | 0 | 114 | 0,8 |
|  | *SC* | *725* | *100* | *0* | *0* | *0* | *0* |
|  | O | 0 | 0 | 0 | 0 | 67 | 100 |
|  | **T** | **573** | **92,1** | **0** | **0** | **49** | **7,9** |
| **3** | Control | 1285 | 88,8 | 67 | 4,6 | 95 | 6,6 |
|  | *SC* | *3429* | *65,8* | *0* | *0* | *1783* | *34,2* |
|  | O | 17188 | 100 | 0 | 0 | 0 | 0 |
|  | **T** | **1803** | **85,7** | **87** | **4,1** | **214** | **10,2** |
| **4** | Control | 1 | 100 | 0 | 0 | 0 | 0 |
|  | *SC* | *1761* | *100* | *0* | *0* | *0* | *0* |
|  | O | 112 | 100 | 0 | 0 | 0 | 0 |
|  | **T** | **192** | **68,6** | **0** | **0** | **88** | **31,4** |
| **5** | Control | 609 | 100 | 0 | 0 | 0 | 0 |
|  | *SC* | *650* | *100* | *0* | *0* | *0* | *0* |
|  | O | 16558 | 100 | 0 | 0 | 0 | 0 |
|  | **T** | **184** | **90,6** | **2** | **1** | **17** | **8,4** |
